# Clinical Trials Have Gone Global: Is This a Good Thing?

**DOI:** 10.1371/journal.pmed.1001228

**Published:** 2012-06-12

**Authors:** Trudie Lang, Sisira Siribaddana

**Affiliations:** 1 Centre for Tropical Medicine, University of Oxford, Oxford, United Kingdom; 2 Faculty of Medicine & Allied Sciences, University of Rajarata, Mihintale, Sri Lanka; 3 Institute of Research & Development, Battaramulla, Sri Lanka

## Abstract

As part of a cluster of articles leading up to the 2012 World Health Report and critically reflecting on the theme of “no health without research,” Trudie Lang and Sisira Siribaddana discuss the value and challenges of doing clinical trials in developing countries.

**Summary Points**

- Clinical trials are conducted across the globe for perfectly good reasons. This is positive because populations in developing countries are under-represented in research.
- Research sites in developing countries benefit from working with externally sponsored clinical trials because they benefit from increased capacity development and investment.
- Locally led research is becoming harder to undertake in developing countries because of complex trial regulations and administrative burdens. There should be a balance between local and externally led trials.
- There is a need for more trials that compare different approaches to managing disease and health issues. This is especially true in low-income settings where simple interventions could make significant improvements to health outcomes if there was evidence to support implementation.
- Clinical trials operations should be specific to the risk and complexity of each trial and not governed by one-size-fits-all requirements of sponsors and their contracted organisations. Overly burdening trials with too-rigorous requirements is pushing up costs and putting off investigators to undertake research.
- Trials in low-income settings need to contribute to clinical trial methodology research efforts.

*In anticipation of the 2012* World Health Report, *this paper was commissioned to help contextualize and critically reflect on the theme of “no health without research.”*

## Why Do We Need Trials and What Makes a Trial a Trial?

Clinical trials are needed globally to reduce disease burdens by helping developing safe and effective new therapies and vaccines. These solutions may be for non-communicable diseases like cancer and diabetes, or, as is especially needed in the poorest regions of the world, infectious disease. Developing countries are under-represented in research due to lack of commercial viability and trained researchers, yet it is in these poorest regions where research-led solutions could bring the greatest impact to high rates of early mortality.

As a research tool clinical trials are fundamental in the effort to develop new products by gaining the data required by regulators, whether for product license extensions for existing therapies for common ailments or to bring cutting edge new therapies and vaccines into approved use. However, there is also a need for clinical trials to bring evidence to determine how to improve the management of health issues; these studies often do not involve a medicinal product but instead compare different options, such as different types of management of an illness in hospital with community-based care. Or, for example, a clinical trial might be used to assess different mechanisms to improve patient adherence to therapy. These pragmatic disease management trials can bring about significant improvements in public health and often require large yet simple trial designs.

The World Health Organization and journal editors define clinical trials as “any research study that prospectively assigns human participants or groups of humans to one or more health-related interventions to evaluate the effects on health outcomes” [1]. Patients may be randomised to an intervention involving either an investigational new product or the standard-of-care treatment, or the patient might be randomised to be cared for by nurses who have been trained in one of two or more comparative ways.

## Why Go Global?

Clinical trial data are often collected from varied populations to support a license application because geographically different trial sites are needed to ensure the product is safe and works in the same way in varying ethnic groups. This requirement is true whether it is a pharmaceutical company working on the next blockbuster drug or a non-for-profit partnership (which typically have a pharmaceutical partner involved in a non-for-profit capacity) developing a new drug or vaccine for a neglected disease. Here scientific and regulatory factors combine to encourage the globalisation of clinical trials.

Clinical trials are also being conducted across more diverse countries for economic reasons. Clinical trials are expensive and are taking longer to conduct than in the past, thus further compounding the increased costs [2], and this is the case for all types of trial, whether commercial or academic. There are many reasons for the increased cost and duration of clinical trials, but it is a widely held view that clinical regulations, or more precisely, the interpretation and implementation of these regulations, are a major factor [2]. Few would argue with the importance of well-regulated clinical trials to ensure high ethical standards and that trial conduct and processes are producing valid and accurate data. However, there is a call for making trial regulation less complicated and more readily adaptable to risk, and for having guidelines that are globally applicable and adaptable to all types of trial [3],[4]. Such guidelines would be as easily applied to pragmatic trials of existing treatments or disease management questions as they would be for trials of new drugs and vaccines.

There are many justifiable reasons for running clinical trials across multiple countries and indeed continents, or even only in sites that are not in the sponsor's location. Some countries are able to recruit participants faster than others for varied and valid reasons. The trial could be for a rare health event, such as dengue fever or traumatic cerebral hemorrhage, and for these trials it is necessary to recruit many centers in diverse locations, each site perhaps recruiting just a few patients to avoid prolonging the duration of the trial and increasing the wait for lifesaving new interventions. It is also true that some regions of the globe are vastly more expensive than others to conduct trials. For example, a clinical trial in India can cost one-tenth of the price that it would cost in the US [5]. Since clinical trials costs are largely driven by labour, much of these savings are from lower salaries to physicians, nurses, and trial coordinators. The time and cost of developing drugs or vaccine influences the final product cost and return on investment, so the logic of reducing trial costs is clear and reasonable.

## What Are the Ethical, Scientific, and Operational Challenges of Running Trials across the Globe?

Clinical trials should be designed, conducted, and monitored in proportion to their relative risk and complexity [6]. However, in developing countries it is our experience that external sponsors and their locally appointed contract research organisations (CROs) are often overly zealous in their interpretation of trial guidelines and apply a one-size-fits-all approach to trial coordination and monitoring, irrespective of the risk and complexity. This is often due to an inaccurate perception from the sponsor and/or CRO about the ability of the research sites to run high quality and compliant trials. This perspective can lead to overly cumbersome trials and to burdening of research sites with administrative requests and site visits that are not necessary. In addition, steps and processes are introduced that can alter the expectations of ethics committees, funders, and reviewers and become perpetuated, irrespective of the real need. Many research teams in developing countries do not have the experience to question the necessity of these overly stringent requirements, which therefore remain in place and become the expected norm for every clinical research study.

For example, some ethics committees insist on Data Safety and Monitoring Committees being put in place for every trial even if these committees are not needed or appropriate [7]. However, trials groups often comply without challenging the request, and the requirement becomes a standard step in the process, without examining each time whether it is appropriate for the specific protocol in question. We are not suggesting in any way that processes and standards should be lower or different in developing countries, but we do feel that overly cautious application of regulations is common around the world- and that it creates a greater burden to research in resource-limited settings.

Table 1 provides examples of steps and issues in clinical trial conduct that could be adversely impacted by inappropriate interpretation of guidelines or overly keen CROs and sponsors. These steps are important for optimum trial conduct and should be carefully considered for each trial and its specific risks and complexity. However, overly “cooking” or implementing these steps can unnecessarily overburden and increase the cost of clinical trials, which results in slowing new product development and discourages investigators from conducting their own independent research.

**Table 1:** Areas of clinical trial conduct where overly cautious application of guidelines (“guideline application creep”) might be problematic.

Areas of Clinical Trial Conduct	Issues, Risks, or Cautions	Advisory Notes
Clinical trial monitoring	It is essential that steps are put in place to ensure that clinical trials collect quality data and are run to high ethical standards.	Trial monitoring should depend upon the risk and complexity of the trial and not be a “one-size-fits-all” application of the common perception of “full” clinical trial monitoring.
	In some situations (often external sponsored trials) monitoring is disproportionately zealous relative to the nature of the trial and is unnecessarily burdensome on the trial site.	The aim of a monitoring or quality management plan should be to ensure the question is being answered accurately and the rights and safety of the patients are protected. All approaches can be assessed against this pragmatic purpose.
	In other circumstances (often academic trials) there is not enough monitoring to ensure the data is being captured accurately and that the participants' rights and safety are being protected.	
	There are many misconceptions about what is needed and these fuel the above two issues of over- and under-monitoring.	In low-income countries and other resource-limited settings highly effective alternatives to commercial monitoring are a very good solution. Examples include training in-house monitoring and reciprocal monitoring between research sites.
Data safety boards	Sponsors and review boards are requesting DSMBs for all clinical trials.	DSMBs are very important in many trials, such as blinded trials where the question may be answered before the end of the trial, or if there is any risk from an intervention.
	Putting a DSMB in place where they are not needed increases the cost of the trial and takes the time of DSMB members where their expertise is in short supply, especially in developing countries.	Some trials do not need a DSMB, such as very fast or open trials, or trials with a low risk intervention.
		In developing countries there is a lack of researchers with the appropriate skills and experience to be members of these committees, which is even more reason to carefully consider whether they are needed.
Clinical trial laboratories	Some sponsors are insisting on laboratory accreditation and this is driving the belief from investigators that there are some very arduous and expensive requirements for clinical trial laboratories.	Laboratory measurements taken for use in clinical trial need to be accurate and reliable. Simple measures and processes can assure this.
	Insisting on accreditation could push regulators in regions to take the position that this is the norm and necessary and insist on accredited laboratories for all trials.	Whilst positive for those that have it, accreditation is not necessary and working toward Good Clinical Trial Laboratory Practices is readily and inexpensively achievable.
	This will further inhibit locally led research and increase the costs for everyone.	In developing countries laboratories are working together to achieve international clinical trial standard laboratory practices. This is easily done by laboratories sharing quality standards, templates, and operating procedures.
Assent from children	In many countries seeking assent from children to take part in clinical trials is a legal requirement.	There is a need from multi-region social science and ethnographic research into the appropriateness and effectiveness of seeking assent from children to take part in clinical research.
	This may not be appropriate or meaningful where autonomy for children is not normal; it is especially important to consider this in developing countries.	Investigators should undergo community engagement to assess what is locally appropriate and acceptable. Then sponsors and regulators should respect these findings.
Clinical trial training	It is important that all those working on a clinical trial are appropriately trained so that they understand the protocol and the trial procedures to ensure consistency, compliance with the protocol, and high standards	There are no specific requirements or qualifications for investigators or indeed clinical trial trainers.
	Some sponsors and regulatory agencies insist on specific training or accredited trainers.	Sponsors of trials need to ensure that investigators have the appropriate experience and qualifications, but there are no minimum requirements.
	Similar to the situation with the laboratories, there are no specific requirements for training or trainers. Misconceptions about this are discouraging locally led trials and raising the expectations of review committees.	Clinical trial training should be given by those with the appropriate experience and training. This does not need to be someone external to the research site or an outside contractor.
		Good Clinical Practice training is important for everyone involved in a trial. Again this can be provided within research sites by an experienced clinical trialist and does not need to involve expensive organisations. There are also plenty of high-quality free online courses available.
		Trial networks in many low-income countries collaborate to share training between their research sites; this is a very effective and a credible mechanism for increasing capacity.

DSMB, data safety and monitoring board.

In the US and the UK it is increasingly recognised that trials have become too expensive and bureaucratic, and initiatives such as the Clinical Trial Transformation Initiative [8] and the Medical Research Council's Methodology Research Hubs in the UK are trying to rationalise design, conduct, and regulation to improve clinical trial design and make running them easier, more attractive, and less expensive. There is a danger that developing and middle-income countries are not involved in this emerging enthusiasm and effort to make trial design more rational and attractive to potential researchers.

It is essential to protect participants in clinical trials from exploitation and this needs particular care and thought in developing countries where populations can be more vulnerable. To achieve this participant protection, great efforts have been made in recent years to strengthen ethical and regulatory review in developing countries, which is, of course, extremely necessary and important. However, whilst in high-income countries efforts have been made to streamline and simplify ethics and the regulatory review process, application to ethics committees has become highly administrative in resource-limited countries with increasing paper work, and multiple sequential reviews are often needed before a trial can start. This administrative hurdle unfortunately further discourages local academic researchers in developing countries [9]. More wealthy foreign trial sponsors may well have the capacity to resource the administrative burden of getting protocols through these committees; however, low investment and support for ethics and regulatory committees in developing countries is a problem for external sponsors. When international product development efforts are delayed by slow review of trial protocols, this seriously increases the time it takes to develop new drugs and vaccines for diseases of poverty such as tuberculosis and malaria.

## What Do We Need to Do Differently?

Clinical trials have gone global and this is certainly a good thing—on the whole. Conducting varying types of trials in low- and middle-income countries (LMICs) can be very positive and the experience research sites gain by working with commercial or not-for-profit sponsors raises research standards and brings health improvements to developing countries and badly needed investment to these research institutions. Externally sponsored trials also bring increasing capacity for research through training and engagement in product development and other global public health initiatives.

However, the global research community needs to improve efforts to support and encourage investigators from LMICs to seek to run their own trials. They need to be provided with incentives and a mandate (possibly from their ministries of health and employing institutions) to plan their own studies and opportunities to diversify beyond externally sponsored trials. A good example of a local investigator-led clinical trial is a recent study in Sri Lanka that addressed a locally relevant question—how to treat snakebites. Interestingly it was advice from a journal's statistician that helped the investigators demonstrate a life-saving intervention to prevent allergic reactions to anti-venom serum for snakebites, which is now being widely used [10]. It is an example of a pragmatic trial that used existing therapies to solve a local issue. The fact that external statistical support was needed shows how capacity is limited and wide support and collaboration are important.

Widespread disparities in clinical care, scientific and health literacy, and economic and social development exist between developed and developing countries. These differences carry concerns about exploitation exacerbated by the power gap between patient-participants and physician-investigators. The vulnerability of developing world patient-participants has been discussed extensively in the past decade [11].

When running trials in vulnerable populations, such as rural communities in developing countries, detailed consideration needs to be given to engaging with the community, explaining the research that is planned, and then carefully selecting the most appropriate approaches for seeking fully informed consent [12]. More social science-based research is needed to make sure that the best approaches, messages, and methods are being practiced in order to protect the study population (and wider community) and also to ensure that the message is being clearly explained and understood. Do those giving consent really understand what is being asked of them? Do they understand that they have a choice; that they are taking part in research and this differs from standard care and, most importantly, that they can say no?

Clinical trial methodology research is needed because there are great differences in cultures and perceptions across the globe, and what is appropriate in one place might not be in another, and so it might not be appropriate to simply export a requirement, and again for this requirement to become introduced unchecked into becoming a standard requirement. For example seeking assent from children is a legal requirement in many countries. Is it always appropriate to apply this rule everywhere? Should a child be asked for assent when they do not normally have any autonomy?

There is a need for training and support for clinical trial investigators and their teams, as well as a need for strengthening capacity for scientific and ethical review in these regions. This capacity needs to be cross-cutting and not focused on one disease or protocol if it is to leave trial sites with the skills and knowledge to run their own studies [13]. New globally appropriate guidelines for good clinical practice would greatly benefit researchers working in non-investigational product trials irrespective of where they are in the world. These guidelines need to be informed by internationally based methodology research.

Risk and complexity-based assessment of trials would improve trial conduct, reduce costs, and enable key elements such as quality management to be more likely to pick up real issues that impact trial outcomes, rather than the one-size-fits-all approach to clinical trial monitoring (often described as “tick box checking”) [14].

We feel that pre-trial community engagement, ongoing dialogue, and post-trial information giving are important to build and foster community trust for clinical research. Researchers in the developing world should come from the same or similar community and relative standard of living in which the research is being done. Not only would mean they have a sense of belonging to that community and the country, but the country and the community also would own and take pride in their researchers. This relationship will only work if the community receives and perceives tangible and intangible benefits from research. Post-trial access to medicines and devices are an integral part of this creation of trust between researchers and the community. In the case of two clinical trials in Sri Lanka, for example, (one for a snakebite treatment and the other a treatment for yellow oleander poisoning [15],[16]), the products are not available locally because the costs are too high.

In addition we all need to be watchful about exporting mistakes made in the northern hemisphere. Whilst the US and Europe are examining how to encourage more academic trials and limit bureaucracy, these same problems are being applied with extra vigour in less experienced settings. We have found that when there is limited experience, those individuals tasked with reviewing research opt for caution and ask for more rather than less. Whilst this situation is correct and understandable, it highlights the need for research reviewers in developing countries to be better supported and provided with the knowledge and confidence to know which requirements to be applied and when. The current excess of caution is limiting research and making trials more expensive and complex than they need to be. The ramifications are important; too few academic trials and the slow development of new drugs and vaccines in regions of the world most burdened by disease directly impact efforts to reduce early mortality in diseases of poverty.

Finally, the globalisation of clinical trials should not be about running inexpensive trial sites to benefit distant people, but should focus on bringing research to populations who have previously been under-represented in clinical trials, and enabling these same communities the benefits resulting from new drugs, vaccines, and improvements in managing health.
